# Efficacy and Safety of Monoclonal Antibodies in the Treatment of HER2-Positive Breast Cancer: A Systematic Review

**DOI:** 10.3390/cancers18213577

**Published:** 2026-11-06

**Authors:** Lucía Alfaro-Muñoz, Ana Avedillo-Salas, Jorge Vicente-Romero, Ana Fanlo-Villacampa

**Affiliations:** 1 Department of Pharmacology, Physiology and Legal and Forensic Medicine, Faculty of Medicine, University of Zaragoza, 50009 Zaragoza, Spainjorgevr@unizar.es (J.V.-R.);; 2 Research Network on Global Evidence on Drug Regulation and Safety (GEDRAS), Institute for Health Research Aragón, Faculty of Medicine, University of Zaragoza, 50009 Zaragoza, Spain

**Keywords:** atezolizumab, bevacizumab, denosumab, HER2-positive breast cancer, monoclonal antibodies, pertuzumab

## Abstract

**Background/Objectives**: HER2-positive breast cancer is one of the most aggressive subtypes of breast cancer, characterised by overexpression of the HER2 receptor, which promotes tumour proliferation and is associated with a poorer clinical prognosis. The introduction of targeted therapies based on monoclonal antibodies has brought about a significant change in the management of the disease, improving survival and disease control. However, the efficacy and safety of some of these treatments remain under investigation. The aim was to evaluate the efficacy and safety of monoclonal antibodies in adult patients with HER2-positive breast cancer compared with placebo or any other drug, with both treatment and control groups receiving other anticancer therapies. **Methods**: A systematic review of the scientific literature was conducted, based on phase III, randomized, controlled, double-blind clinical trials published between 2020 and 2026 in the PubMed, Cochrane and Scopus databases. The review was carried out following the PICO framework and the PRISMA guidelines. **Results**: Eight reports of seven clinical trials were included, evaluating four monoclonal antibodies: pertuzumab, denosumab, atezolizumab and bevacizumab. Treatment with pertuzumab showed a significant improvement in tumour response and progression-free survival with an acceptable safety profile. In contrast, bevacizumab, denosumab and atezolizumab did not show significant clinical benefits and, furthermore, were associated with a higher incidence of adverse effects. **Conclusions**: Pertuzumab is an effective therapeutic strategy for the treatment of HER2-positive breast cancer. However, individualised treatment selection and appropriate monitoring of adverse effects are necessary.

## Simple Summary

HER2-positive breast cancer is an aggressive subtype in which HER2 overexpression promotes tumor growth and increases the risk of disease recurrence. Monoclonal antibodies have improved treatment outcomes, although their efficacy and safety vary across therapeutic strategies. The aim of this systematic review was to evaluate the efficacy and safety of monoclonal antibodies in adults with HER2-positive breast cancer. We identified eight reports of seven phase III randomized controlled trials evaluating four monoclonal antibodies: pertuzumab, denosumab, atezolizumab, and bevacizumab. Pertuzumab, particularly combined with trastuzumab, improved tumor response and progression-related outcomes with an acceptable safety profile. Denosumab and atezolizumab showed no significant clinical benefit, whereas bevacizumab did not improve efficacy and increased toxicity. Overall, these findings highlight the heterogeneous efficacy and safety profiles of monoclonal antibodies and support treatment selection according to the clinical setting and individual patient characteristics.

## 1. Introduction

### 1.1. Breast Cancer

Breast cancer is the most commonly diagnosed cancer among women worldwide, with approximately 2.3 million new cases in 2020, and represents a major cause of cancer-related morbidity and mortality among women [1]. The global burden of the disease continues to increase, with the number of new cases projected to rise substantially in the coming decades. Persistent disparities in early detection and access to effective treatment further contribute to differences in outcomes between populations [1,2,3,4,5,6].

Breast cancer is a heterogeneous disease comprising different histological and molecular subtypes with distinct biological characteristics, prognoses, and responses to treatment. Tumours are assigned to receptor-based clinical groups (surrogate subtypes) according to hormone receptor and human epidermal growth factor receptor 2 (HER2) expression: hormone receptor-positive/HER2-negative, HER2-positive, and triple-negative. These groups approximate, but are not identical to, the intrinsic molecular subtypes (luminal A, luminal B, HER2-enriched and basal-like) defined by gene-expression profiling. HER2-positive breast cancer accounts for approximately 15–20% of breast cancers and is characterised by HER2 protein overexpression and/or ERBB2 gene amplification [7,8,9]. Accurate determination of HER2 status is essential for treatment selection and is routinely performed using immunohistochemistry and/or in situ hybridisation according to established diagnostic criteria [8,10].

HER2 is a transmembrane receptor tyrosine kinase belonging to the ErbB receptor family. Its activation promotes tumour-cell proliferation and survival through intracellular signalling pathways, particularly the phosphatidylinositol 3-kinase/protein kinase B (PI3K/AKT) and mitogen-activated protein kinase (MAPK) pathways. HER2 can form heterodimers with other members of the ErbB family, especially HER3, resulting in potent activation of downstream signalling. Persistent activation of these pathways contributes to the aggressive biological behaviour of HER2-positive breast cancer and provides a rationale for targeted therapeutic intervention [11,12].

### 1.2. Therapeutic Options

The treatment of breast cancer is individualised according to tumour stage, molecular subtype, biological characteristics, and patient-related factors. Current therapeutic strategies include surgery, radiotherapy, chemotherapy, endocrine therapy, immunotherapy, and molecularly targeted treatments [13,14,15,16].

Surgery and radiotherapy constitute the principal local treatments for patients with non-metastatic disease, whereas systemic therapy is selected according to the risk of recurrence, tumour biology, disease stage, and previous treatment. Chemotherapy remains an important treatment modality for patients with high-risk or aggressive disease, while endocrine therapy is indicated in tumours expressing hormone receptors. Other systemic approaches include immune checkpoint inhibitors and molecularly targeted therapies directed against specific receptors, ligands, or intracellular signalling pathways [13,17,18].

In HER2-positive breast cancer, the identification of HER2 as a therapeutically actionable molecular target led to the development of monoclonal antibodies specifically directed against this receptor [19,20,21,22,23]. Trastuzumab was the first HER2-targeted monoclonal antibody to demonstrate a substantial clinical benefit in patients with HER2-positive breast cancer. The addition of trastuzumab to chemotherapy significantly improved clinical outcomes in metastatic disease and subsequently reduced the risk of recurrence in early-stage disease [24,25,26].

The therapeutic landscape has subsequently expanded beyond direct HER2 blockade. Monoclonal antibodies directed against HER2, including pertuzumab and margetuximab, have been developed to provide complementary mechanisms of action. In addition, monoclonal antibodies directed against other biological targets, such as receptor activator of nuclear factor-κB ligand (RANKL), programmed death-ligand 1 (PD-L1), and vascular endothelial growth factor (VEGF), have been investigated in patients with HER2-positive breast cancer because of the involvement of bone remodelling, immune regulation, and tumour angiogenesis in breast cancer progression [19,23,26,27,28].

### 1.3. Monoclonal Antibodies in HER2-Positive Breast Cancer

Monoclonal antibodies represent an important group of biological therapies investigated in HER2-positive breast cancer. These agents differ substantially in their molecular targets and mechanisms of action. Trastuzumab and pertuzumab directly target HER2, whereas margetuximab combines HER2 binding with Fc engineering intended to enhance immune-mediated antitumour activity. Pertuzumab binds to a different extracellular domain of HER2 from trastuzumab and interferes with receptor dimerization, particularly HER2–HER3 heterodimerization. Several studies demonstrated that the addition of pertuzumab to trastuzumab and docetaxel significantly improved progression-free and overall survival in patients with previously untreated HER2-positive metastatic breast cancer [27,28].

Margetuximab represents a further development of HER2-directed antibody therapy. This Fc-engineered monoclonal antibody was designed to enhance interaction with Fcγ receptors and potentially increase antibody-dependent cellular cytotoxicity [23,29]. In addition, margetuximab combined with chemotherapy significantly improved progression-free survival compared with trastuzumab plus chemotherapy in patients with previously treated HER2-positive advanced breast cancer, although the final overall survival analysis did not demonstrate a difference between treatment groups [30,31].

Other monoclonal antibodies have also been investigated in HER2-positive breast cancer despite having targets other than HER2. Denosumab is a fully human monoclonal antibody directed against RANKL, a key mediator of osteoclast differentiation and bone resorption. Its use in breast cancer has primarily been investigated in the context of bone health and skeletal complications, particularly in patients receiving systemic anticancer treatment [32,33]. Atezolizumab, an anti-PD-L1 monoclonal antibody, has been evaluated as an immunotherapeutic strategy based on the role of immune checkpoint signalling in tumour immune evasion [34]. Bevacizumab, which targets VEGF-A, has also been investigated in breast cancer because angiogenesis is an important component of tumour growth and progression [20,35,36].

Therefore, monoclonal antibodies evaluated in HER2-positive breast cancer do not constitute a pharmacologically homogeneous group. Some directly inhibit HER2 signalling, whereas others act on biological pathways that may influence tumour progression, immune responses, or treatment-associated complications. This distinction is relevant when interpreting the efficacy and safety of these therapies and when comparing results across clinical trials.

In addition to monoclonal antibodies, the therapeutic management of HER2-positive breast cancer has been expanded by antibody–drug conjugates (ADCs), such as trastuzumab emtansine and trastuzumab deruxtecan, and by tyrosine kinase inhibitors (TKIs), including lapatinib, neratinib, and tucatinib. ADCs combine antibody-mediated targeting with the delivery of a cytotoxic payload, whereas TKIs inhibit intracellular tyrosine kinase activity. These agents therefore represent pharmacologically distinct therapeutic classes and should be considered separately from unconjugated monoclonal antibodies when evaluating treatment efficacy and safety.

Despite substantial advances in the management of HER2-positive breast cancer, clinically relevant differences remain in the efficacy and safety of monoclonal antibody therapies. These differences may depend on the molecular target, treatment setting, disease stage, line of treatment, combination regimen, previous exposure to systemic therapy, and patient and tumour characteristics. Furthermore, monoclonal antibodies may be associated with clinically relevant adverse events, including cardiac toxicity, infusion-related reactions, gastrointestinal toxicity, haematological abnormalities, and immune-mediated adverse events, depending on their mechanism of action [24,25,27,28,37].

## 2. Justification and Aims

HER2-positive breast cancer is an aggressive subtype associated with increased tumour proliferation, disease recurrence and higher mortality. Although the development of targeted therapies has substantially improved clinical outcomes, disease progression and treatment-related toxicity remain significant clinical challenges. Monoclonal antibodies have expanded the therapeutic options available to these patients; however, differences in their mechanisms of action, efficacy and safety profiles make it necessary to systematically evaluate the available evidence.

Therefore, the main objective of this systematic review is to assess the efficacy and safety of monoclonal antibodies used in adult patients with HER2-positive breast cancer, compared with placebo or conventional treatments, based on clinical trials published from 2020 to 2026.

## 3. Materials and Methods

### 3.1. Search Strategy

The search was conducted using PubMed, Cochrane and Scopus. The studies were identified by combining the names “HER2” and “breast cancer” and “randomized controlled trial” with the keywords monoclonal antibody, pertuzumab, atezolizumab, margetuximab, bevacizumab, denosumab and their drug class. MeSH (Medical Subject Headings) terms and the Boolean operators “AND” and “OR” were also used in the search (Supplementary Information Table S1).

**Figure 1:**
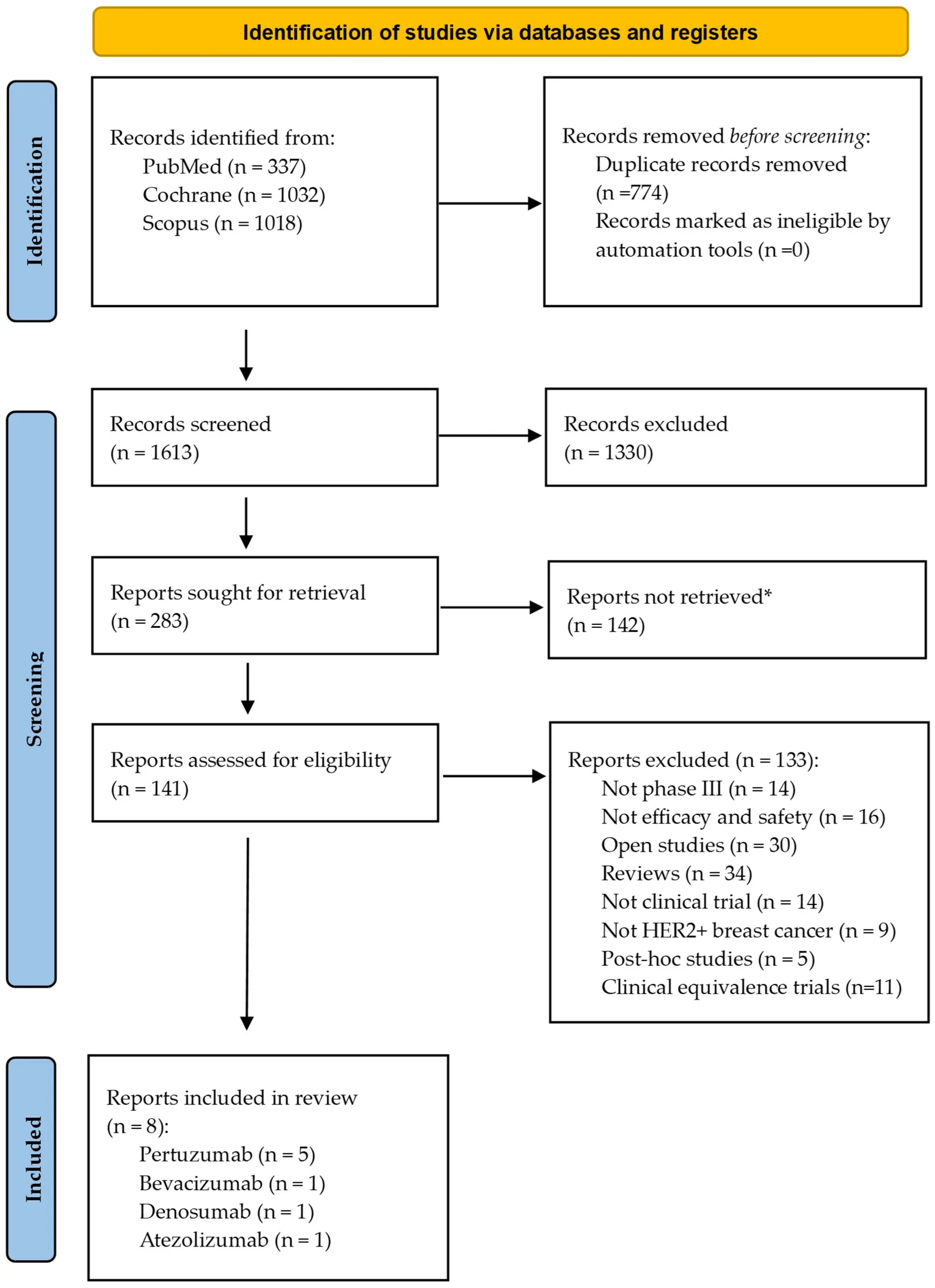
PRISMA flow diagram illustrating the process of selecting studies included in the systematic review [38]. * Full text unavailable.

This review was performed in accordance with the PRISMA (Preferred Reporting Items for Systematic Reviews and Meta-Analyses) guidelines and has not been registered [38]. Studies published from 2020 to 2026 were identified and retrieved. Following duplicate removal, titles and abstracts were screened to exclude records that did not fulfil the predefined inclusion criteria. The full texts of the remaining studies were subsequently examined to determine their eligibility for inclusion in the review. The selection process is summarized in Figure 1.

### 3.2. Inclusion Criteria

Eligible studies were phase III, double-blind, randomized controlled trials. The remaining eligibility criteria were established in accordance with the PICO framework, as summarized in Table 1.

### 3.3. Exclusion Criteria

The exclusion criteria for this systematic review were as follows: (a) studies providing insufficient data for analysis; (b) in vitro, in silico, or animal studies; (c) commentaries, expert opinions, case reports, letters to the editor, narrative or systematic reviews, meta-analyses, study protocols, study registry records, and observational studies; (d) phase I or phase II clinical trials; (e) studies in which none of the monoclonal antibodies of interest was evaluated as an intervention, including studies focusing on antibody–drug conjugates, bispecific antibodies, or tyrosine kinase inhibitors as therapeutic interventions; (f) studies investigating medicinal plants; (g) clinical equivalence studies comparing monoclonal antibodies with their reference biologic products; and (h) studies involving pregnant or breastfeeding women.

### 3.4. Data Collection and Analysis

Data extraction was performed independently by three reviewers (A.A.-S., A.F.-V., and J.V.-R.). The following information was collected from each eligible trial: (a) clinical trial registration number; (b) author(s); (c) year of publication; (d) study design; (e) participant characteristics; (f) intervention details, including dosage regimens; (g) comparator treatment and its dosage regimen; (h) participants’ age; and (i) study outcomes. The extracted information was compiled in an Excel spreadsheet and independently cross-checked by three authors (L.A.-M., A.F.-V., and J.V.-R.). Any discrepancies arising during data extraction were discussed and resolved with the involvement of the senior author (A.A.-S.). Reports of the same trial were linked to a single unique study, and participants were counted only once.

### 3.5. Quality Assessment and Risk of Bias

The quality of evidence was assessed following the Grading of Recommendations Assessment, Development and Evaluation (GRADE) system [39] (Supplementary Information Table S2). Risk of bias was assessed at trial level with the Cochrane Risk of Bias 2 (RoB 2) tool, and the domain-level judgements, with their supporting rationale (Supplementary Information Figure S1).

## 4. Results

Eight reports corresponding to seven unique randomized, double-blind phase III trials were included: PEONY (two reports), PUFFIN, CLEOPATRA, APHINITY, D-CARE, IMpassion050 and E1105.

### 4.1. Pertuzumab

Pertuzumab was evaluated in four phase III, randomized, double-blind, placebo-controlled clinical trials: PEONY, PUFFIN, CLEOPATRA and APHINITY. These studies investigated the efficacy and safety of pertuzumab in combination with trastuzumab and docetaxel in patients with HER2-positive breast cancer across different disease settings.

PEONY trial evaluated in this study on the basis of two publications: Shao et al. [40] and Huang et al. [41] included 329 Asian women with HER2-positive early or locally advanced breast cancer. Patients with tumors > 2 cm and T2–3 or T4 disease were randomly assigned in a 2:1 ratio to receive neoadjuvant pertuzumab, trastuzumab, and docetaxel (n = 219) or placebo, trastuzumab, and docetaxel (n = 110). The experimental group received four cycles of intravenous pertuzumab (840 mg loading dose followed by 420 mg), trastuzumab (8 mg/kg loading dose followed by 6 mg/kg), and docetaxel (75 mg/m^2^) every 3 weeks before surgery. Following surgery, patients received adjuvant chemotherapy and anti-HER2 therapy for a total treatment duration of one year.

Shao et al. [40] evaluated pathological and clinical response. In the intention-to-treat population, total pathological complete response rate (tpCR) was significantly higher with pertuzumab than with placebo (39.3% vs. 21.8%), corresponding to an absolute difference of 17.5 percentage points (95% CI: 6.9–28.0; *p* = 0.001). The objective response rate was also higher in the pertuzumab group (88.6% vs. 78.2%), whereas complete clinical response rates were similar between groups. These findings indicate a higher pathological and objective response with dual HER2 blockade (Table 2).

The overall incidence of adverse events (AEs) was comparable between groups. The most frequently reported AEs were alopecia, neutropenia, leukopenia, and diarrhea, with diarrhea occurring more frequently in patients receiving pertuzumab (38.5% vs. 16.4%). Serious AEs occurred in 10.1% of patients in the pertuzumab group and 8.2% in the control group. No clinically relevant cardiac events or treatment-related deaths were reported.

Huang et al. [41] evaluated long-term efficacy in the PEONY population using event-free survival (EFS), disease-free survival (DFS), and overall survival (OS). At 5 years, EFS was higher with pertuzumab than with placebo (84.8% vs. 73.7%; HR: 0.53, 95% CI: 0.32–0.89; *p* = 0.027). Similarly, 5-year DFS was 86.0% versus 75.0%, respectively (HR: 0.52, 95% CI: 0.30–0.88; *p* = 0.028). Five-year OS also numerically favored pertuzumab (93.9% vs. 90.0%), although the difference did not reach statistical significance (HR: 0.53, 95% CI: 0.23–1.19).

The safety profile remained broadly comparable between treatment groups. The most common AEs were neutropenia, leukopenia, alopecia, nausea, and diarrhea, with diarrhea again occurring more frequently with pertuzumab (40.8% vs. 17.3%). Serious AEs were reported in 17.0% and 13.6% of patients, respectively. Treatment-related deaths were uncommon (0.9% vs. 1.8%), and no clinically relevant cardiac events were observed. A reduction in left ventricular ejection fraction (LVEF) to <40% was reported in 0.9% of patients in each group.

PUFFIN trial analyzed by Xu et al. [42] included 243 Chinese patients with previously untreated locally recurrent or metastatic HER2-positive breast cancer. Patients were randomly assigned in a 1:1 ratio to receive pertuzumab, trastuzumab, and docetaxel (n = 122) or placebo, trastuzumab, and docetaxel (n = 121). Pertuzumab was administered intravenously at a loading dose of 840 mg followed by 420 mg every 3 weeks, together with trastuzumab (8 mg/kg loading dose followed by 6 mg/kg) and docetaxel (75 mg/m^2^ every 3 weeks). Anti-HER2 treatment was continued until disease progression or unacceptable toxicity.

Xu et al. [42] evaluated progression-free survival (PFS) and OS, with tumor response and disease progression assessed according to RECIST 1.1 criteria. In the intention-to-treat population, median PFS was longer with pertuzumab than with placebo (16.5 vs. 12.5 months), corresponding to a significant reduction in the risk of progression or death (HR: 0.60, 95% CI: 0.45–0.81; *p* = 0.0008). OS also favoured pertuzumab, although statistical significance was not reached (HR: 0.68, 95% CI: 0.45–1.03; *p* = 0.0658) (Table 2).

AEs of any grade were reported in 99.2% of patients receiving pertuzumab and 95.8% of controls, while serious AEs occurred in 24.6% and 19.2%, respectively. The most frequent events were leukopenia and neutropenia, followed by anemia, alopecia, and diarrhea. Diarrhea was more frequent in the pertuzumab group (45.9% vs. 23.3%). Treatment-related deaths occurred in 1.7% of patients receiving pertuzumab and 3.3% of placebo. No cases of heart failure or clinically significant reductions in LVEF to <40% were observed.

CLEOPATRA trial evaluated by Swain et al. [43] included 808 patients with previously untreated metastatic HER2-positive breast cancer, randomly assigned in a 1:1 ratio to pertuzumab, trastuzumab, and docetaxel (n = 402) or placebo, trastuzumab, and docetaxel (n = 406). Pertuzumab was administered intravenously at a loading dose of 840 mg followed by 420 mg every 3 weeks, together with trastuzumab (8 mg/kg loading dose followed by 6 mg/kg) and docetaxel (75 mg/m^2^, with escalation to 100 mg/m^2^ permitted if tolerated). Pertuzumab or placebo and trastuzumab were continued until disease progression or unacceptable toxicity, whereas docetaxel was administered for at least six cycles and could be continued at the investigator’s discretion.

Swain et al. [43] evaluated long-term OS and PFS. In the intention-to-treat population, median OS was substantially longer with pertuzumab than with placebo (57.1 vs. 40.8 months), corresponding to a 16.3-month difference and a reduced risk of death (HR: 0.69, 95% CI: 0.58–0.82). At 8 years, the OS rate was 37% with pertuzumab compared with 23% with placebo, indicating sustained long-term benefit from dual HER2 blockade. Investigator-assessed PFS was also prolonged with pertuzumab (18.7 vs. 12.4 months; HR: 0.69, 95% CI: 0.59–0.81; *p* < 0.0001) (Table 2).

The overall safety profile was broadly comparable between groups. The most frequent grade 3–4 AE was neutropenia, occurring in 49% of patients in the pertuzumab group and 46% of controls. Diarrhea was more frequent with pertuzumab. Treatment-related deaths occurred in five patients (1%) receiving pertuzumab and six patients (2%) receiving placebo. No major clinically relevant cardiac safety signal was observed.

APHINITY trial included 4.805 patients after excision of invasive HER2-positive with node-positive or high-risk node-negative breast cancer. Patients were randomly assigned in a 1:1 ratio to receive treatment with pertuzumab a loading fixed dose of 840 mg intravenously, then a fixed dose of 420 mg once every 3 weeks for 1 year in combination with trastuzumab, a loading dose of 8 mg/kg of body weight intravenously then at 6 mg/kg once every 3 weeks for 1 year (corresponding to 18 cycles) and adjuvant chemotherapy (n = 2.400) or placebo, trastuzumab, and adjuvant chemotherapy (n = 2.405) for 1 year. Piccart et al. [44] in a second interim analysis (median follow-up 6 years) evaluated efficacy according to overall survival (OS) and invasive disease-free survival (IDFS) in APHINITY. The analysis of OS did not show statistically significant differences between the two treatment groups (HR: 0.85, 95% CI: 0.67–1.07; *p* = 0.17). At 6 years, the invasive disease-free survival (IDFS) was 91% with pertuzumab versus 88% with placebo remain event-free (95% CI: 1.0–4.6). The clinical benefit was primarily observed in patients with node-positive disease (HR 0.72, 95% CI 0.59–0.87), whereas no clear advantage was found in those with node-negative disease, where the IDFS rate was 95% across both treatment arms (Table 2).

With regard to safety, the incidence of primary cardiac events was low (0.8 per cent in the pertuzumab group versus 0.3 per cent in the placebo group), as was the incidence of secondary cardiac events (2.7 per cent in the pertuzumab group versus 2.8 per cent in the placebo group), with one additional case of heart failure recorded in the pertuzumab group. There were two deaths related to a cardiac condition in the pertuzumab group and four in the placebo group.

### 4.2. Denosumab

Denosumab was evaluated in this systematic review in the D-CARE phase III trial, reported by Coleman et al. [45]. This international, multicenter, randomized, double-blind, placebo-controlled trial investigated whether inhibition of the RANKL could improve clinical outcomes in patients with high-risk stage II or III breast cancer.

A total of 4509 patients with stage II or III breast cancer and an ECOG performance status of 0–1 were randomly assigned in a 1:1 ratio to receive denosumab or placebo in addition to standard treatment, including chemotherapy, endocrine therapy, and/or anti-HER2 therapy, as clinically indicated. Denosumab was administered subcutaneously at a dose of 120 mg every 3–4 weeks for approximately 6 months, followed by maintenance administration every 12 weeks, for a total treatment duration of 5 years.

Coleman et al. [45] evaluated efficacy primarily according to bone metastasis-free survival (BMFS), as well as DFS and OS. In the intention-to-treat population, no significant difference in BMFS was observed between denosumab and placebo (HR: 0.97, 95% CI: 0.82–1.14; *p* = 0.70). Similarly, DFS was comparable between groups (HR: 1.04, 95% CI: 0.91–1.19), with no evidence of a clinical benefit from the addition of denosumab to standard treatment. No significant differences were observed in OS or other secondary endpoints, and no patient subgroup with a clinically relevant benefit was identified (Table 3).

In terms of safety, the overall incidence of AEs was broadly comparable between the denosumab and placebo groups. The most frequently reported AEs included nausea, arthralgia, alopecia, fatigue, and diarrhea. The most common serious or grade ≥ 3 hematological events were neutropenia (15% vs. 15%), febrile neutropenia (5% vs. 6%), and leukopenia (3% vs. 3%). However, denosumab was associated with a higher incidence of osteonecrosis of the jaw (5% vs. <1%) and hypocalcemia (7% vs. 4%). No treatment-related deaths were reported in the denosumab group, whereas two occurred in the placebo group.

### 4.3. Atezolizumab

Atezolizumab, a monoclonal antibody directed against programmed death-ligand 1 (PD-L1), was assessed in the present systematic review through the phase III IMpassion050 trial published by Huober et al. [46]. This randomized, double-blind, placebo-controlled study evaluated the efficacy and safety of incorporating atezolizumab into standard neoadjuvant chemotherapy alongside dual HER2-targeted therapy in patients with high-risk, early-stage HER2-positive breast cancer.

A total of 454 patients with high-risk disease, defined by a primary tumour > 2 cm and node-positive disease (cT2–4, cN1–3, M0), were randomly assigned 1:1 to receive atezolizumab or placebo in combination with neoadjuvant dose-dense doxorubicin and cyclophosphamide followed by paclitaxel, together with trastuzumab and pertuzumab. Following surgery, atezolizumab or placebo was planned to continue with anti-HER2 therapy until the completion of one year of HER2-targeted therapy (patients with residual disease could switch to ado-trastuzumab emtansine with atezolizumab or placebo). Planned follow-up, not treatment, extended up to 5 years. The randomized study treatment was discontinued early following an unfavourable benefit–risk assessment.

Huober et al. [46] primarily evaluated efficacy according to the pathological complete response (pCR) rate. In the intention-to-treat population, pCR rates were similar between patients receiving atezolizumab and placebo (62.4% vs. 62.7%), with an absolute difference of −0.33 percentage points (95% CI: −9.23 to 8.57; *p* = 0.9551). In the PD-L1-positive subgroup, pCR was numerically lower with atezolizumab than with placebo (64.2% vs. 72.5%), although the difference was not statistically significant (absolute difference: −8.26%; 95% CI: −20.56 to 4.04; *p* = 0.1846) (Table 3). Results were consistent across the other prespecified subgroups, with no evidence of an improvement in pathological response following the addition of atezolizumab to dual HER2 blockade and chemotherapy.

Regarding safety, AEs were frequent in both treatment groups. The most commonly reported events included fatigue, vomiting, rash, and hepatic abnormalities, while immune-mediated events such as hypothyroidism and hepatitis were more frequent with atezolizumab. During the neoadjuvant phase, grade 3–4 AEs occurred in 51.8% of patients receiving atezolizumab compared with 43.6% of those receiving placebo, while overall serious AEs were reported in 19.5% and 13.3%, respectively. Five grade 5 (fatal) adverse events occurred, all in the atezolizumab arm (four in the neoadjuvant and one in the adjuvant phase); two were attributed to study treatment.

### 4.4. Bevacizumab

Bevacizumab was evaluated in this systematic review in the E1105 phase III trial, reported by Mezzanotte-Sharpe et al. [47]. This randomized, double-blind, placebo-controlled trial investigated the efficacy and safety of adding bevacizumab to standard chemotherapy and trastuzumab in patients with HER2-positive metastatic breast cancer.

This trial included patients aged ≥18 years with HER2-positive metastatic breast cancer, an ECOG performance status of 0–1, and no previous systemic treatment for metastatic disease. A total of 96 patients were randomly assigned in a 1:1 ratio to receive first-line chemotherapy and trastuzumab combined with bevacizumab or placebo; 93 patients initiated study treatment. During the 24-week induction phase, patients received paclitaxel (90 mg/m^2^ weekly or 80 mg/m^2^ in combination with carboplatin) plus trastuzumab (4 mg/kg loading dose followed by 2 mg/kg weekly) and bevacizumab (10 mg/kg every 2 weeks) or placebo. Thereafter, maintenance treatment consisted of trastuzumab (6 mg/kg every 3 weeks) combined with bevacizumab (15 mg/kg every 3 weeks) or placebo until disease progression or unacceptable toxicity.

Mezzanotte-Sharpe et al. [47] primarily evaluated efficacy according to PFS, ORR, and OS, as well as health-related quality of life assessed using FACT/FACIT questionnaires. In the intention-to-treat population, median PFS was numerically longer with bevacizumab than with placebo (13.8 vs. 11.1 months), although the difference was not statistically significant (HR: 0.73, 95% CI: 0.43–1.23; *p* = 0.24). The ORR was also higher with bevacizumab (61% vs. 54%), while median OS was 63.0 versus 49.1 months, respectively (HR: 1.09, 95% CI: 0.61–1.97; *p* = 0.75). However, neither ORR nor OS showed a statistically significant difference between treatment groups (Table 3).

Regarding safety, the most frequently reported AEs in both groups were neutropenia and hypertension. Left ventricular dysfunction, fatigue, and sensory neuropathy were more frequently reported with bevacizumab. Grade toxicity ≥3 AEs occurred in 67% of patients receiving bevacizumab compared with 47% receiving placebo. One treatment-related death due to catheter-associated infection was reported in the bevacizumab group. Congestive heart failure, assessed according to the New York Heart Association (NYHA) functional classification, occurred more frequently with bevacizumab than with placebo (4 cases vs. 1 case).

## 5. Discussion

The studies analyzed in this review show that monoclonal antibodies targeting HER2 have represented a significant advance in the treatment of HER2-positive breast cancer, particularly when used in combination with chemotherapy. However, the results reveal marked heterogeneity amongst the therapeutic strategies analyzed, both in terms of clinical benefit and toxicity, with significant differences observed depending on the drug’s mechanism of action, the line of treatment and the clinical context of the disease.

Dual HER2 blockade using pertuzumab and trastuzumab is the strategy with the strongest evidence of efficacy. In the neoadjuvant setting, the PEONY trial [40,41] demonstrated a significant increase in pathological complete response with the addition of pertuzumab to trastuzumab and chemotherapy. Furthermore, long-term follow-up revealed improvements in event-free survival and disease-free survival. These results are clinically relevant because pathological response following neoadjuvant treatment is a marker associated with long-term prognosis in certain subtypes of HER2-positive breast cancer. Furthermore, the results obtained with pertuzumab are consistent with the central role of HER2 in the biology of this subtype of breast cancer. Pertuzumab and trastuzumab have complementary mechanisms of action, as they bind to different extracellular domains of HER2 and interfere with distinct signalling pathways. This complementarity provides a biological basis for the benefit observed with dual blockade in metastatic disease and reinforces the concept that the simultaneous inhibition of HER2 through different HER2-mediated signalling pathways may contribute to more prolonged disease control.

CLEOPATRA trial [43], the addition of pertuzumab to treatment with trastuzumab and docetaxel resulted in a substantial improvement in progression-free survival and overall survival. The benefit observed following long-term follow-up, with a difference in more than 16 months in median overall survival, is particularly significant in the context of metastatic disease. The persistence of the effect in the long term also suggests that the advantage associated with dual blockade is not limited to an initial response, but may translate into sustained disease control. In the adjuvant setting, APHINITY [44] showed an improvement in invasive disease-free survival that was concentrated in node-positive disease, with no clear benefit in node-negative disease and no significant overall survival difference at the interim analysis, which illustrates that the magnitude of the benefit depends on the baseline risk of recurrence. Furthermore, the available evidence indicates that the benefit of anti-HER2 treatment is not necessarily uniform across all patients. Factors such as lymph node involvement, hormone receptor status and disease stage may modify the absolute benefit obtained from intensified therapy. Therefore, the results of this review support an interpretation based on risk stratification rather than the uniform application of intensified treatment strategies.

The results of the PEONY trial [40] demonstrate a significant improvement in the complete pathological response rate in the neoadjuvant setting, which is associated with a better prognosis. Furthermore, long-term results showed a significant improvement in both event-free survival and disease-free survival at 5 years, indicating that the benefit of dual HER2 blockade is not limited to the initial response to treatment, but is sustained over time. These findings are consistent with the results of PUFFIN and CLEOPATRA studies in metastatic disease [42,43], demonstrating a significant reduction in the risk of progression and a clear benefit in overall survival, particularly evident in the CLEOPATRA study with a gain of more than 16 months. Together with APHINITY, these results are consistent with the established role of dual HER2 blockade in the clinical settings in which it is indicated. With regard to safety, pertuzumab demonstrated a favourable profile. Diarrhoea was the most common adverse effect, whilst neutropenia and leucopenia were primarily associated with concomitant chemotherapy. Of particular note is the absence of a significant increase in cardiac toxicity in the trials reviewed, which reinforces the safety of the combined use of anti-HER2 antibodies, even during prolonged treatment.

In contrast, other therapeutic strategies evaluated in this review did not improve their primary efficacy endpoints. Thus, the D-CARE trial [45] shows that RANKL inhibition using denosumab, despite its solid biological basis, did not improve bone metastasis-free survival, disease-free survival or overall survival—all of which are clinically relevant outcomes in early-stage disease. D-CARE was not restricted to HER2-positive disease: HER2 status was a stratification factor and approximately one fifth of participants had HER2-positive tumours. All efficacy estimates reported refer to the overall, mixed-subtype population and therefore constitute indirect evidence for HER2-positive breast cancer; they do not by themselves establish the efficacy of denosumab in this subtype. Furthermore, although the safety profile was acceptable, a higher incidence of mandibular osteonecrosis and hypocalcaemia was observed in the denosumab-treated group, which may impact quality of life and treatment adherence, particularly given the long duration of treatment. These results are particularly relevant as they illustrate the importance of distinguishing between a plausible mechanistic hypothesis and a demonstrated clinical benefit. Although the RANK/RANKL pathway is involved in bone remodelling and has been experimentally linked to processes implicated in tumour progression, the D-CARE trial did not show a significant reduction in bone metastasis-free survival or improvements in disease-free survival or overall survival. Therefore, the available results do not support a systemic antitumour effect of denosumab in this population. However, this conclusion must be distinguished from its usefulness in the prevention or treatment of skeletal complications, which constitutes a separate therapeutic objective.

Similarly, the IMpassion050 study [46] shows that the addition of atezolizumab immunotherapy to standard neoadjuvant treatment does not improve the complete pathological response rate, either in the overall population or in the PD-L1-positive subgroups, and was associated with more grade 3–4 and serious adverse events, including five fatal adverse events in the atezolizumab arm (two attributed to study treatment). These results call into question the current role of immunotherapy in early-stage HER2-positive breast cancer, suggesting that, unlike other subtypes of breast cancer such as triple-negative breast cancer, the addition of immune checkpoint inhibitors may play a more limited role in this type of breast cancer. Furthermore, the increase in serious adverse events and immune-mediated toxicities in the atezolizumab-treated group raises doubts about the benefit-risk ratio of this therapeutic strategy. Longer follow-up of event-free survival is required to inform the long-term role of atezolizumab. The absence of clinical benefit observed on pathological response with atezolizumab is also relevant from the perspective of precision medicine. The rationale for incorporating a PD-L1 inhibitor into the treatment of HER2-positive breast cancer is based on the interaction between HER2, the tumour microenvironment and the immune response. However, in the IMpassion050 trial, the addition of atezolizumab to chemotherapy and dual HER2 blockade did not increase the rate of pathological complete response, not even among patients with PD-L1-positive tumours. These results suggest that PD-L1 expression, when considered in isolation, does not appear sufficient to identify patients most likely to benefit from this strategy. The interaction between tumour immunogenicity, lymphocyte infiltration, HER2 expression and other mechanisms of immune regulation is likely to be more complex.

In the E1105 trial [47], the addition of bevacizumab to standard therapy failed to produce statistically significant improvements in efficacy outcomes. Although progression-free survival, objective response rate, and overall survival showed numerically better results in the bevacizumab group, none of these differences reached statistical significance. Moreover, the relatively small sample size limits the precision of the estimates, warranting cautious interpretation of the findings.

Thus, these results do not provide sufficient evidence that VEGF inhibition offers an additional clinical benefit to HER2 blockade in this population. The absence of a significant improvement in efficacy, coupled with a higher incidence of grade ≥3 AEs—including cardiovascular events such as hypertension, left ventricular dysfunction and congestive heart failure—suggests that its clinical utility is limited in this context.

The endpoints are not interchangeable: pCR and ORR are early response measures; EFS, DFS and IDFS capture recurrence after curative-intent treatment; PFS captures progression in advanced disease; and OS is the most clinically definitive endpoint but is influenced by subsequent therapy.

In the neoadjuvant setting, pertuzumab increased tpCR in PEONY, with improved 5-year EFS and DFS and a non-significant OS difference, whereas atezolizumab did not increase pCR over dual HER2 blockade plus chemotherapy in IMpassion050 (ITT and PD-L1-positive subgroup). In the adjuvant setting, pertuzumab improved IDFS in APHINITY, chiefly in node-positive disease, without a significant OS difference at the interim analysis; the denosumab trial (D-CARE), whose overall-population estimates are indirect for HER2-positive disease, showed no improvement in BMFS, DFS or OS. In first-line metastatic disease, pertuzumab improved PFS in PUFFIN and CLEOPATRA and OS in CLEOPATRA, whereas bevacizumab did not significantly improve PFS, ORR or OS in the underpowered E1105 trial.

Nevertheless, the results of this systematic review should be interpreted within the specific clinical context of each study. The included trials showed considerable heterogeneity in terms of disease stage, patient characteristics, prior lines of treatment, concomitant chemotherapy regimens, outcome variables and duration of follow-up, so the evidence does not support a ranking of the antibodies. These differences limit direct comparability between the different monoclonal antibodies and suggest that the results should be interpreted in light of the therapeutic setting in which they were evaluated. Furthermore, the absence of a significant effect on a given endpoint benefit with a particular antibody does not necessarily imply that its biological target lacks therapeutic relevance; rather, it may reflect the complexity of its interaction with other molecular pathways, concomitant treatments, the statistical power of the trial and specific characteristics of the study population.

This aspect is particularly relevant in the current context of HER2-positive breast cancer, where the therapeutic arsenal has expanded with the development of antibody-drug conjugates and tyrosine kinase inhibitors. In this scenario, monoclonal antibodies should increasingly be regarded as components of sequential, risk-adapted therapeutic strategies, rather than as stand-alone interventions. The prolonged survival observed with strategies based on dual HER2 blockade highlights the impact that complementary and sustained inhibition of this pathway can achieve. However, the introduction of new targeted therapies raises questions regarding the optimal treatment sequence, patient selection and the potential balance between therapeutic intensification and de-escalation.

Overall, the evidence reviewed supports the value of complementary HER2 blockade using trastuzumab and pertuzumab in the clinical contexts studied in which it is indicated, whilst the results obtained with antibodies targeting other molecules demonstrate that the identification of a potentially relevant molecular mechanism does not in itself guarantee an additional clinical benefit over an established anti-HER2 strategy. Therefore, the therapeutic impact of a monoclonal antibody appears to depend on the interaction between the molecular target, the clinical context, the characteristics of the disease and concomitant treatment. Looking ahead to future research, it will be necessary to determine not only which targeted therapies are effective, but also which patients may derive a clinically relevant benefit, at what stage they should be administered, and how they should be optimally integrated with available anti-HER2 treatments. This perspective will be essential for progressing towards more individualised, effective and rational treatment strategies for HER2-positive breast cancer.

This systematic review has several strengths. Firstly, randomized clinical trials were included, thereby prioritising comparative evidence with a high degree of internal validity. Secondly, efficacy and safety were assessed jointly, allowing for consideration of the therapeutic balance of each antibody and not merely its efficacy. Thirdly, the inclusion of studies conducted in both early-stage and metastatic breast cancer provides a broad overview of the clinical applicability of these therapies. However, several limitations must be borne in mind when interpreting the results. The number of trials available for some of the monoclonal antibodies was small, and there was clinical and methodological heterogeneity amongst the studies. Differences in disease stage, line of treatment, chemotherapy regimen, dose, duration of follow-up and definition of outcome variables limited the possibility of making quantitative comparisons between the agents. Likewise, some trials had relatively small sample sizes, such as E1105, which limits the precision of their efficacy estimates. The interpretation of long-term results may also be influenced by subsequent treatments, particularly in metastatic disease. Furthermore, the overall assessment of the evidence using the GRADE framework indicated a high/moderate level of certainty, which must be taken into careful consideration when interpreting the results.

Future studies should focus on identifying biomarkers capable of predicting more accurately which patients may benefit from specific strategies involving monoclonal antibodies. In particular, characterising HER2 expression, hormone receptor status, immunological biomarkers, tumour heterogeneity and resistance mechanisms could contribute to a more individualised therapeutic selection. The negative results observed with atezolizumab on pathological response also suggest that PD-L1 expression alone may be insufficient to identify patients who benefit from immune checkpoint inhibition in this disease. Therefore, future research should evaluate combined biomarkers that integrate tumour and immunological characteristics.

## 6. Conclusions

The results of this systematic review show that the clinical benefit of monoclonal antibodies in HER2-positive breast cancer is heterogeneous and depends on the mechanism of action and the clinical context. Pertuzumab, in combination with trastuzumab and chemotherapy, showed the most consistent evidence of benefit in the neoadjuvant, adjuvant and first-line metastatic settings studied, with improvements in pathological response and survival and an overall acceptable safety profile. Denosumab, atezolizumab and bevacizumab did not demonstrate significant benefits in the primary efficacy endpoints and were associated, to varying degrees, with increased toxicity. Tailoring treatment to the characteristics of the tumour and the patient, as well as of identifying predictive biomarkers, will be crucial to the optimising treatment selection. Further studies are considered necessary to better define the role of these emerging therapies and to obtain long-term data.

## Figures and Tables

**Table 1:** Inclusion criteria based on PICO framework.

P	Patient	Adult patients (>18 years) diagnosed with HER-2 positive breast cancer
I	Intervention	Treatment with monoclonal antibodies
C	Comparison	Placebo, standard care, any other drug treatment
O	Outcome	Efficacy: total pathological complete response rate (tpCR rate), clinical complete response rate, clinical partial response rate, event-free survival rate (EFS rate), disease-free survival rate (DFS rate), invasive disease-free survival (IDFS), progression-free survival (PFS) events, PFS median, overall survival (OS) events, OS median, objective response rate (ORR rate), bone metastasis-free survival (BMFS), pathological complete response (pCR), programmed death-ligand 1 (PD-L1)-positive, PD-L1-negative. Safety: incidence and severity of adverse reactions

CI, confidence interval; DFS, disease-free survival; IDFS, invasive disease-free survival; EFS, event-free survival; HR, hazard ratio; OS, overall survival; PFS, progression-free survival; RR, rate ratio; tpCR, total pathological complete response; y, years.

**Table 2:** Efficacy outcomes of pertuzumab in HER2-positive breast cancer.

	PEONY			PEONY			PUFFIN			CLEOPATRA			APHINITY		
	Shao et al., 2020 [40]			Huang et al., 2024 [41]			Xu et al., 2023 [42]			Swain et al., 2020 [43]			Piccart et al., 2021 [44]		
	Pertuzumab (n = 219)	Placebo (n = 110)	*p*	Pertuzumab (n = 219)	Placebo (n = 110)	*p*	Pertuzumab (n = 122)	Placebo (n = 121)	*p*	Pertuzumab (n = 402)	Placebo (n = 406)	*p*	Pertuzumab (n = 2.400)	Placebo (n = 2.405)	*p*
*tpCR rate*	39.3%	21.8%													
Difference (95% CI)	17.5% (6.9–28.0)		0.001												
RR (95% CI)	1.80 (1.22–2.66)														
*Clinical complete response rate (%)*	11.0	10.0													
*Clinical partial response rate (%)*	77.6	68.2													
*EFS rate, 5-years (%)*				84.8	73.7										
Difference (95% CI)				11.1 (1.2–21.0)		0.027									
*HR* (95% CI)				0.53 (0.32–0.89)											
*DFS rate, 5-years (%)*				86.0	75.0										
Difference (95% CI)				11.0 (1.2–20.7)		0.028									
*HR* (95% CI)				0.52 (0.30–0.88)											
IDFS *rate, 6-years (%)*													91	88	
Difference (95% CI)													(1.0–4.6)		
*HR* (95% CI) (node-positive disease)													0.72 (0.59–0.87)		
PFS															
*PFS events (%)*							68.9	81.8							
*PFS, median (months)*							16.5	12.5		18.7	12.4				
*HR* (95% CI)							0.60 (0.45–0.81)		0.0008	0.69 (0.59–0.81)					
*OS*															
*OS events (%)*							3y: 32.8	41.3		8y: 58	69				
*OS median (months)*										57.1	40.8				
HR (95% CI)							0.68 (0.45–1.03)		0.0658	0.69 (0.58–0.82)					
*OS, 8-years (%)*										37	23				
*OS, 5-years (%)*				93.9	90.0										
Difference (95% CI)				3.9 (2.9–10.7)		0.262									
*HR* (95% CI)				0.53 (0.23–1.19)											
*OS, 6-years (%)*															
*HR* (95% CI)													0.85 (0.67–1.07) 0.17		

CI, confidence interval; DFS, disease-free survival; IDFS, invasive disease-free survival; EFS, event-free survival; HR, hazard ratio; OS, overall survival; PFS, progression-free survival; RR, rate ratio; tpCR, total pathological complete response; y, years.

**Table 3:** Efficacy outcomes of denosumab, atezolizumab and bevacizumab in HER2-positive breast cancer.

**Reference/Study**	**Population**	**Intervention**	**Control**	**Sample Size, n**	**Treatment Duration/Follow-Up**	**Efficacy Outcomes**
**Coleman et al., 2020 [45] D-CARE** (NCT01077154)	4509 women with stage II–III high-risk breast cancer ^a^	Denosumab + chemotherapy	Placebo + chemotherapy	2256/2253	5 years	BMFS: HR 0.97 (95% CI, 0.82–1.14), *p* = 0.70. DFS: HR 1.04 (95% CI, 0.91–1.19), *p* = 0.57. OS: HR 1.03 (95% CI, 0.85–1.25), *p* = 0.76.
**Huober et al., 2022 [46] IMpassion050** (NCT03726879)	454 patients with high-risk early-stage HER2-positive breast cancer, tumor > 2 cm and positive lymph nodes	Atezolizumab + dose-dense doxorubicin/cyclophosphamide followed by paclitaxel + trastuzumab + pertuzumab	Placebo + dose-dense doxorubicin/cyclophosphamide followed by paclitaxel + trastuzumab + pertuzumab	226/228	Treatment until completion of 1 year of anti-HER2 therapy	pCR (ITT): 62.4% vs. 62.7%; difference −0.33% (95% CI, −9.23 to 8.57), *p* = 0.9551. pCR in PD-L1-positive patients: 64.2% vs. 72.5%. pCR in PD-L1-negative patients: 60.7% vs. 53.8%.
**Mezzanotte-Sharpe et al., 2024 [47] E1105** (NCT00520975)	96 patients with HER2-positive metastatic breast cancer who had not previously received systemic treatment for metastatic disease	Bevacizumab + trastuzumab + paclitaxel ± carboplatin	Placebo + trastuzumab + paclitaxel ± carboplatin	48/48	Induction: 24 weeks (6 cycles), followed by maintenance until disease progression, toxicity, or death	Median PFS: 13.8 vs. 11.1 months; HR 0.73 (95% CI, 0.43–1.23), *p* = 0.24. ORR: 61% vs. 54%. Median OS: 63.0 vs. 49.1 months; HR 1.09 (95% CI, 0.61–1.97), *p* = 0.75. Complete response: 13% vs. 8%. Partial response: 48% vs. 46%.

**BMFS**, bone metastasis-free survival; **CI**, confidence interval; **DFS**, disease-free survival; **HER2**, human epidermal growth factor receptor 2; **HR**, hazard ratio; **ITT**, intention-to-treat; **ORR**, objective response rate; **OS**, overall survival; **PFS**, progression-free survival; **pCR**, pathological complete response; ^a^ immunohistochemical subtypes of breast cancer: hormone receptor-positive and HER2-negative.

## Data Availability

No new data were created or analyzed in this study. Data sharing is not applicable to this article.

## Supplementary Materials

The following supporting information can be downloaded at https://www.mdpi.com/article/10.3390/cancers18213577/s1, Table S1: Search Strategy; Table S2: GRADE for domains associated with the efficacy and safety of monoclonal antibody treatment in HER2-positive breast cancer; Table S3: PRISMA 2020 Checklist; Figure S1: Risk of bias of the articles included in the systematic review.
